# Efficacy of a foamed disinfectant in reducing pathogen contamination in renovated inpatient in-room sinks: a randomized controlled trial

**DOI:** 10.1017/ice.2025.10318

**Published:** 2026-01

**Authors:** Bobby Glenn Warren, Amanda M. Graves, Guerbine Fils-Aime, Aaron Barrett, Isadora Mamikunian, Claudia Gunsch, Becky A. Smith, Deverick J. Anderson

**Affiliations:** 1 Disinfection, Resistance, and Transmission Epidemiology (DiRTE) Lab, Durham, NC, USA; 2 Duke Center for Antimicrobial Stewardship and Infection Prevention, Division of Infectious Diseases, Duke University Medical Center, Durham, NC, USA; 3 Department of Civil and Environmental Engineering, Duke University, Durham, NC, USA

## Abstract

**Background:**

Hospital sinks are reservoirs for epidemiologically important pathogens (EIPs), yet practical, effective strategies for sustained decontamination are lacking.

**Methods:**

We conducted a randomized controlled trial of 30 in-room sinks (15 intervention, 15 control) in a newly renovated hospital unit to evaluate the efficacy of a hydrogen peroxide/peracetic acid foamed disinfectant in reducing sink contamination. Intervention sinks received foamed disinfectant to sink drains three times weekly; control sinks underwent standard daily surface cleaning. Weekly sampling was performed from three sink locations (top surface, tail pipe, P-trap) over 35 weeks. The primary outcome was sink conversion events (SCEs), defined as first detection of ≥1 EIP, defined as *Pseudomonas aeruginosa*, *Stenotrophomonas spp*., or *Acinetobacter* spp., and ESBL-producing or carbapenem-resistant Enterobacterales, in previously negative sinks.

**Results:**

A total of 2880 samples were collected. All sinks were negative at baseline for study pathogens. Nearly all sinks (29/30) experienced an SCE during the study period. However, only 44 (9%) intervention sink samples were positive for EIPs, compared to 270 (47%) in control sinks (*p* < 0.00001). EIPs were recovered from 4% versus 24% of P-traps and 4% versus 39% of tail pipes; sink top/handle contamination was rare and similar (3% vs 4%). The most frequent EIPs were *Acinetobacter* spp. and *Stenotrophomonas* spp. Intervention sinks experienced a delayed time to SCE (*p* = 0.0001). Items were stored on/in sinks in 93% of observations.

**Conclusion:**

Regular application of a foamed disinfectant reduced and delayed EIP contamination in renovated hospital sinks. Foam-based protocols may help mitigate environmental reservoirs of multidrug-resistant organisms.

## Manuscript

## Introduction

Healthcare-associated infections (HAIs) remain a persistent challenge in hospitals, with an estimated 700,000 cases and 75,000 deaths occurring annually in the United States.^1,2^ Although hand hygiene among healthcare personnel has been the primary focus of infection prevention, increasing evidence implicates the hospital environment as an important reservoir and source of transmission for epidemiologically important pathogens (EIPs).^3,4^ Outbreaks linked to water sources and wastewater drains, including hospital sinks, are being reported with increasing frequency in both acute care and intensive care settings.^5–13^

Sinks have become a recognized point of vulnerability within patient rooms. In a recent multicenter study conducted in Germany, intensive care units (ICUs) with in-room sinks had significantly higher HAI incidence compared with units that lacked sinks, and in-room sink presence was an independent risk factor for HAIs.^14^ Despite these findings, removing sinks from patient care may be unrealistic in most U.S. hospitals, given the demands of hand hygiene, workflow, and infection prevention, although some European ICUs have reported success with sink removal strategies. As a result, multiple strategies have been investigated to reduce the risk of sink-related contamination, including the use of liquid chemical disinfectants, installation of antimicrobial metals, and application of steam or ozone, among others. However, these approaches often suffer from high cost, logistical complexity, or limited effectiveness, particularly when applied in real-world clinical environments.^15–23^

Furthermore, the design of modern sink plumbing poses additional challenges for disinfection. The configuration of the P-trap leads to continuous dilution of liquid disinfectants and prevents adequate contact with the tail pipe, the section of plumbing between the drain cover and the P-trap, making thorough disinfection difficult.

Foamed disinfectants have been proposed as a novel intervention to increase the contact time of disinfectant chemicals within the complex geometry of sink plumbing. Recent studies have demonstrated the disinfection efficacy of foamed disinfectants in reducing biofilm burden within sink tail pipes; however, no studies have evaluated the impact of foamed disinfectants on the contamination of p-trap fluid or the top of the sink.^24,25^

Given these challenges, we aimed to assess the effectiveness of a foamed disinfectant intervention, with particular attention to the potential advantages of foaming action for improving sink hygiene and reducing the burden of EIPs in inpatient in-room sinks.

## Methods

### Objective

The primary objective of this study was to evaluate the efficacy of a foamed disinfectant, (Virasept, Ecolab; 3.13% hydrogen peroxide, 0.05% peracetic acid), in reducing contamination of EIPs in inpatient in-room sinks.^26^

### Patient consent statement

This study was reviewed by the Duke University Health System Institutional Review Board and received an “exempt” status.

### Study setting and design

We performed this RCT on a renovated general medicine unit at Duke University Hospital, a 1048-bed tertiary care hospital in Durham, North Carolina. The unit was outfitted with new distal plumbing and individual in-room handwashing sinks prior to the study period. These sinks were standard porcelain basins with limited countertop surface area for storage and did not include splash guards. The unit underwent renovation between June 2023 and June 2024. The unit began admitting patients in July 2024. Initial study samples were collected one week prior to patient occupancy to obtain baseline contamination data. Overall, the study period spanned from July 2024 to March 2025.

All in-room sinks on the study unit were enrolled and randomized in a 1:1 ratio to either the intervention or control arm. The intervention arm received targeted drain disinfection in addition to standard environmental cleaning, while the control arm only received standard environmental cleaning practices. All the clinical, laboratory, and statistical staff were blinded to the randomization; however, the study team members who applied the disinfectant were not blinded to the study group randomization.

### Study protocol

In sinks in the intervention arm, a study team member applied the foamed disinfectant to sink drains three times per week (Monday, Wednesday, and Friday). Foam was dispensed directly into the drain until it filled up to the drain cover and was left to self-dissipate. The control arm received standard hospital cleaning procedures defined as daily surface disinfection with non-bleach solutions performed by environmental services staff. Standard cleaning and disinfection protocols do not include specific treatment of tail pipes or p-traps.

Microbiological samples of the sink were taken once per week throughout the study period. All sampling was conducted at least 24 hours after the last foam application to minimize immediate suppression effects. Samples were obtained from 3 locations from each study sink: the top of the bowl, the tail pipe, and the p-trap. The sample from the top of the bowl included the horizontal surface surrounding the sink bowl as well as the sink handles and was sampled using a cellulose sponge pre-moistened in neutralizing buffer. The sample from the tail pipe included the internal surface of the pipe below the sink drain cover and was taken with a flocked swab pre-drenched in neutralizing buffer. The sample from the p-trap included an agitated liquid sample and was collected by inserting sterile tubing down the drain and using a sterile syringe to repeatedly and vigorously agitate the liquid, thereby dislodging biofilm from the walls of the P-trap.^12^

At each sampling event, all items discovered in or on the in-room sinks were systematically recorded including any clinical equipment, personal belongings, or miscellaneous objects present at the time of sampling. The purpose of this documentation was to identify potential sources of contamination or vectors for pathogen transmission associated with sink use.

### Microbiological methods

Microbiological samples obtained via cellulose sponge were processed using the stomacher technique in accordance with the Centers for Disease Control and Prevention protocol.^27^ Samples taken via swab or syringe were processed by vortexing for 10 seconds. 200 μL of sampled eluent was cultured on HardyCHROM ESBL agar and on cetrimide agar for *Pseudomonas aeruginosa* and *Stenotrophomonas maltophilia* and incubated at 37 °C for 24 hours. Antibiotic susceptibility testing was performed following the Clinical and Laboratory Standards Institute (CLSI) guidelines.^28^ All isolates were speciated using 16S rRNA sequencing. EIPs were defined as *Pseudomonas aeruginosa*, *Stenotrophomonas maltophilia*, *Acinetobacter spp.,* and ESBL- and/or carbapenem-resistant Enterobacterales.

### Outcomes

The primary outcome was a sink conversion event (SCE), defined as the first detection of ≥1 species-specific EIP in a sink where that EIP had not previously been identified. Secondary outcomes included: 1) the frequency and diversity of EIPs recovered from sinks in each study arm, 2) the identification of patient clinical cultures positive for study-defined EIPs and concordance between patient and sink isolates from the same room, and 3) the frequency and nature of items found in or on in-room sinks during the study period. Concentrations of EIPs were not recorded, as the study was performed under time-sensitive conditions and designed to capture presence/absence of pathogens as the primary outcome.

### Statistical analysis

Time-to-event data for SCEs were analyzed using Kaplan-Meier survival curves, with statistical significance assessed by the log-rank test. Proportions of EIPs recovered from sinks between study arms were compared using z-score population tests. All analyses were performed using SAS version 9.4M7 (SAS Institute Inc).

## Results

A total of 30 in-room handwashing sinks were enrolled and randomized; 15 were assigned to the intervention arm and 15 to the control arm. Weekly sampling was performed 32 times over the 35-week study period. The first sampling event, or baseline sampling, was performed one week before the unit opened to patients. No EIPs were detected at baseline sampling. The subsequent 31 sampling events occurred after patients returned. Samples were taken from each sink in 3 locations resulting in a total of 2880 microbiological samples, including 1440 from each study arm and 960 from each sink sample location.

### Primary outcome: sink conversion events (SCEs)

**Figure 1.**
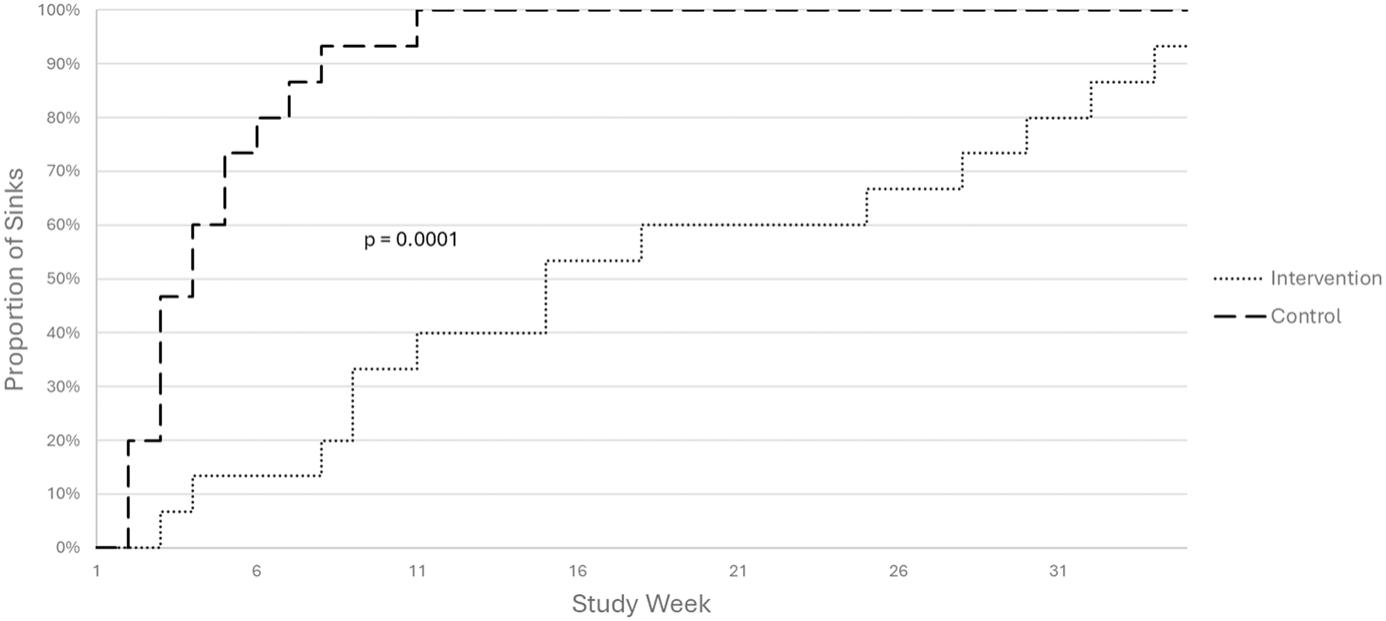
Time to sink conversion event (SCE) with an epidemiologically important pathogen (EIP) by study arm.

We observed 29 SCEs during the 9-month study period: 14 in the intervention arm and 15 in the control arm. In the intervention arm, all 14 SCEs occurred in the P-trap. In the control arm, 11 of the 15 sinks had at least one SCE in the tail pipe and 4 had an SCE in the P-trap. The first SCE detected was from the second sampling event, study week 2, which occurred one week following the unit opening to patients in the control arm. The first SCE in the intervention arm occurred on the third sampling event. By study week 11, all 15 sinks in the control arm (100%) had experienced an SCE, whereas only 6 sinks (40%) in the intervention arm had undergone an SCE. Overall, the intervention arm remained free of EIP contamination for a longer duration than those in the control arm (log-rank *p* = 0.0001; Figure 1). These analyses were repeated at the sample location level and yielded similar results (data not shown).

### Sink contamination by epidemiologically important pathogens (EIPs)

**Figure 2.**
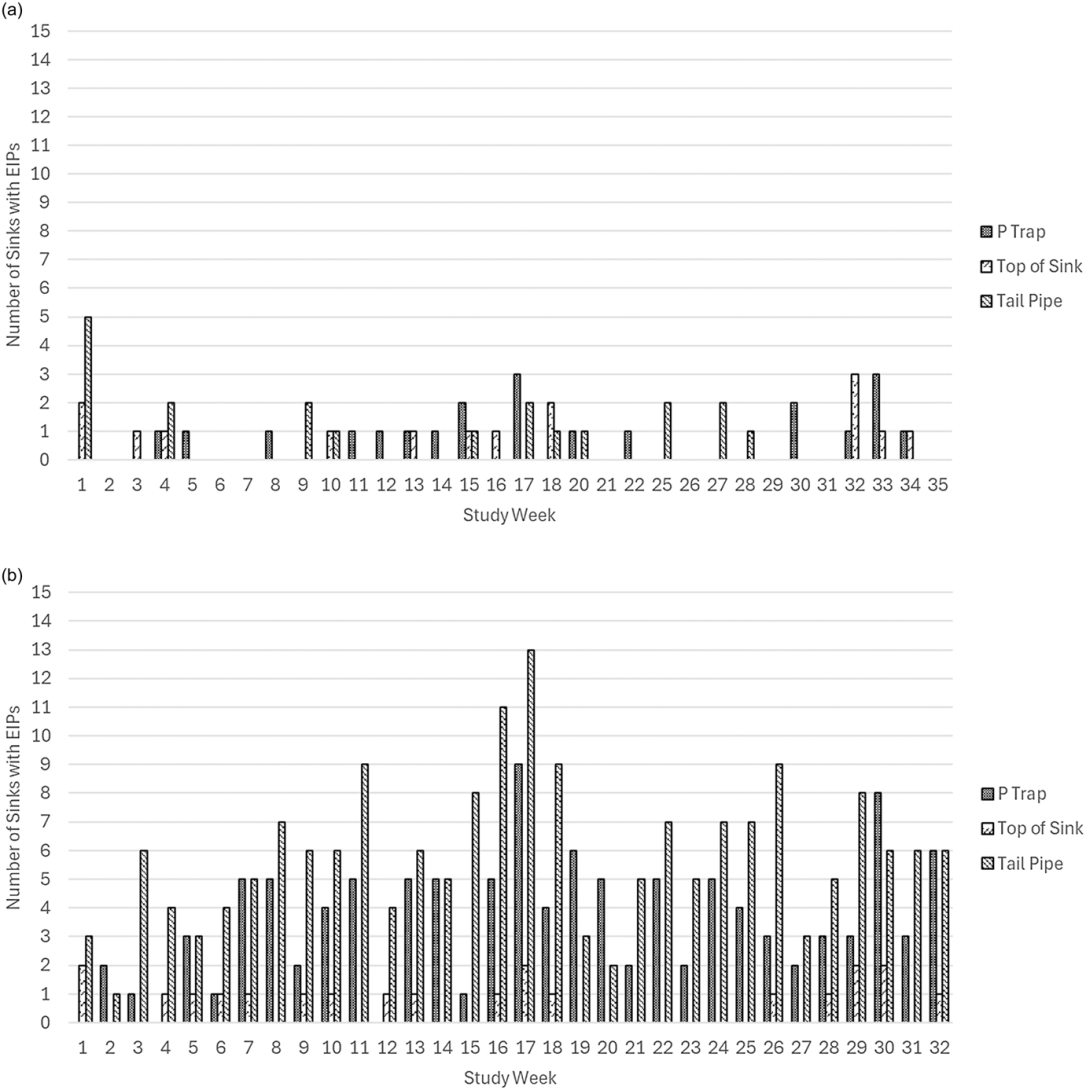
(a) Number of intervention sinks with recovered EIPs over time by sample location. (b) Number of control sinks with recovered EIPs over time by sample location.

Of 960 sink samplings, 270 (28%) yielded EIPs from at least one sample location on a study sink: 44 (9%) in the intervention arm and 226 (47%) in the control arm (*p* < 0.00001). At the sample location level, results for P-trap and tail pipe were consistent with the overall findings. EIPs were recovered from 21 (4%) P-trap samplings and 20 (4%) tail pipe samplings in the intervention arm, compared to 114 (24%) and 189 (39%) in the control arm, respectively, (both *p* < 0.00001). In contrast, there was no significant difference for the top of the sink, with EIPs detected in 15 (3%) intervention samplings and 21 (4%) control samplings (*p* = 0.31). The distribution of EIPs recovered by study arm, sample location, and study week is illustrated in Figure 2. No patients housed in the study unit had matching EIPs in their clinical cultures before or after SCEs during the study, likely reflecting the selective nature of the study’s EIP definition.

### Sink contamination by epidemiologically important pathogen (EIP) genera

Overall, 498 EIPs were recovered from study sinks; 433 (87%) from control sinks, and 65 (13%) from intervention sinks (*p* < 0.01). All EIPs were recovered less frequently in the intervention arm compared to control. However, this difference was not statistically significant for *Burkholderia spp.* and *Citrobacter spp.,* likely due to small numbers. The two most frequent EIPs in both arms were *Acinetobacter spp.* and *Stenotrophomonas spp*. (Table 1).

**Table 1.** Epidemiologically important pathogens recovered from study sinks by study arm and sink sample location

	Overall				Control				Intervention				Intervention versus control *P*-values			
Epidemiologically important pathogen	Total	P-trap	Sink Top	Tail Pipe	Total	P-trap	Sink Top	Tail Pipe	Total	P-trap	Sink Top	Tail Pipe	Total	P-trap	Sink Top	Tail Pipe
*Acinetobacter spp.*	185	65	21	99	157	58	10	89	28	7	11	10	< 0.01	< 0.01	0.77	< 0.01
*Burkholderia spp.*	1	0	0	1	1	0	0	1	0	0	0	0	0.32	1	1	0.16
*Citrobacter spp.*	9	5	0	4	9	5	0	4	0	0	0	0	0.64	< 0.01	1	< 0.01
*Enterobacter spp.*	64	23	1	40	63	22	1	40	1	1	0	0	< 0.01	< 0.01	0.16	< 0.01
*Klebsiella spp.*	33	8	8	17	24	5	4	15	9	3	4	2	< 0.01	0.32	1	< 0.01
*Pseudomonas aeruginosa*	48	26	4	18	41	20	4	17	7	6	0	1	< 0.01	< 0.01	< 0.01	< 0.01
*Serratia spp.*	5	2	0	3	5	2	0	3	0	0	0	0	< 0.01	0.05	1	0.01
*Stenotrophomonas spp.*	153	50	4	99	133	40	4	89	20	10	0	10	< 0.01	< 0.01	< 0.01	< 0.01
Grand total	498	179	38	281	433	152	23	258	65	27	15	23	< 0.01	< 0.01	0.07	< 0.01

179 EIPs were recovered from p-trap samples; 27 (15%) from intervention sinks and 152 (85%) from control sinks (*p* < 0.01). 38 EIPs were recovered from sink top samples; 23 (61%) from intervention sinks and 15 (39%) from control sinks (*p* = 0.07). 281 EIPs were recovered from tail pipe samples; 23 (8%) from intervention sinks and 258 (92%) from control sinks (*p* < 0.01) (Table 1).

### Sink hygiene

Study sinks were evaluated for the presence of items in or on them 810 times, including 393 intervention sink observations and 417 control sink observations. Overall, 756 (93%) observations noted at least one item in or on the study sink. In the intervention arm, 367 (93%) observations noted at least one item in or on the study sink. Similarly, in the control arm, 389 (93%) observations noted at least one item in or on the study sink.

During sink evaluations, a total of 602 items were found in or on study sinks. The most frequently observed category was hygiene-related items, with 336 instances (56%), including items such as bath wipes and wash basins. Medical supplies were the next most common, accounting for 152 items (25%). Food items, such as trays and cups, were found 72 times (12%). Linen and apparel such as pillows, pillowcases, and socks were observed 25 times (4%). Medical devices including spirometers, IV pumps, and thermometers were observed 9 times (1%). An additional 8 items (1%) could not be categorized and included trash bags, flowers, and phones.

## Discussion

In this randomized controlled trial, application of a foamed disinfectant to in-room sinks in a renovated inpatient unit was associated with a marked reduction in contamination by EIPs and a prolonged time to first contamination event, compared to standard cleaning protocols. The intervention arm demonstrated a substantially lower proportion of sink samplings with EIPs, as only 9% of intervention sink samplings were positive compared to 47%. While burden was lower in the intervention arm, nearly all sinks eventually experienced at least one conversion event. These results support the effectiveness of the foamed disinfectant in reducing the overall burden and frequency of EIP contamination but suggest that additional intermittent interventions may be needed to prevent overall conversion events.

The intervention’s effect was most pronounced in the P-trap and tail pipe. For both locations, only 4% of intervention sink samples were positive, versus 24% and 39% in the control arm, respectively. In contrast, there was no significant reduction in contamination of sink tops or handles, and dispersal outside the drainpipes may represent a more clinically important pathway for pathogen transmission. Importantly, all intervention-arm SCEs occurred exclusively in the P-trap, while the tail pipe remained consistently protected, whereas most control sinks developed contamination in the tail pipe. These findings are notable given the challenges in achieving effective disinfection of the tail pipe where residual water and biofilm can persist. To our knowledge, this is the first study to evaluate the impact of a foamed disinfectant on the contamination of both p-trap fluid and the top of the sink in a real-world clinical environment.

A notable aspect of our study was the consistent presence of items in or on study sinks throughout the observation period. We found that 93% of all sink assessments documented at least one item present, including hygiene products, medical supplies, food items, linens, and personal belongings. Hygiene-related items were the most frequently observed, accounting for over half of all objects. The prevalence of such items highlights ongoing challenges in promoting appropriate sink use and underscores the potential for indirect contamination or cross-transmission events. The storage of clinical equipment and supplies in or near sinks can facilitate the transfer of pathogens from contaminated plumbing surfaces to patient care items, thereby increasing the risk for healthcare-associated infections.^28^ These findings emphasize the need for not only environmental interventions, such as enhanced disinfection, but also targeted behavioral and educational strategies to address inappropriate sink use and reduce opportunities for environmental cross-contamination in patient care areas.

Our findings build off previous research evaluating foamed disinfectants for sink drain decontamination. A prior study by Donskey et al. demonstrated that application of the same disinfectant we studied could significantly reduce the total burden of Gram-negative bacteria in hospital sink drains, with near-complete suppression achieved after daily application for seven days.^29^ However, that study assessed only overall Gram-negative counts and reported that all drains were colonized with *Pseudomonas aeruginosa* at baseline, without differentiating specific multidrug-resistant pathogens. In contrast, our study focused on EIPs—specifically *Pseudomonas aeruginosa*, and *Acinetobacter* spp. and ESBL-producing or carbapenem-resistant Enterobacterales. While both studies support the efficacy of foam-based disinfection for reducing sink colonization, our results extend these findings to a more clinically relevant group of pathogens.

A study by Buchan et al. evaluated the effect of a single application of hydrogen peroxide foam versus bleach on bacterial counts in hospital sink drains.^16^ In that study, any bacteria detected were counted, rather than focusing on specific pathogens or resistance patterns. While hydrogen peroxide foam achieved a greater initial reduction in bacterial counts at 24 hours compared to bleach, all sinks returned to baseline levels by day 7. Despite methodological differences, both studies share the finding that hydrogen peroxide foam can produce an initial decrease in sink bacterial contamination.

However, it is important to note that both prior studies were conducted in hospital units with existing clinical use and established biofilm in the plumbing. In contrast, our study was performed in a newly renovated unit with entirely replaced distal plumbing, meaning the sinks had not yet developed substantial biofilm prior to intervention. This difference in baseline conditions may have contributed to a higher observed efficacy of the foamed disinfectant in our study.

This study has many strengths, including its randomized design, blinded outcome assessment, and real-world setting. The use of multiple sampling sites within the sink allowed for a more comprehensive assessment of contamination, while systematic recording of items in and on sinks provided insight into environmental risk factors. This study also has several limitations. First, culture-based sink surveillance may underestimate the true prevalence of EIPs, as detection can be influenced by factors such as the presence of soap, clinical waste, or other fluids in the sink. To improve recovery of contaminating organisms, we specifically agitated the P-trap fluid during sampling to help dislodge biofilm from the walls using previously validated methods.^12^ Second, our study was conducted at a single center and in newly renovated sinks which allowed us to observe the natural history of colonization and the effect of a foamed disinfectant under controlled conditions. However, this design limits generalizability to hospitals where sinks and plumbing have been in long use and biofilm is well established. Third, we did not perform molecular typing to assess clonal relatedness between sink isolates. Finally, microbiological sampling was performed once per week which likely affected the ability to capture granularity of sink contamination during the study.

Our findings have practical implications for infection prevention in healthcare settings. The substantial reduction in EIP contamination observed with the foamed disinfectant, particularly in plumbing locations inaccessible to routine cleaning, suggests that such interventions could be valuable in high-risk environments such as intensive care units or hematology/oncology wards. In parallel, the near-universal presence of items in or on sinks in both study arms underscores the ongoing need for behavioral interventions and education regarding proper sink use.

In conclusion, this randomized controlled trial demonstrated that regular application of a foamed disinfectant to in-room sinks significantly reduced contamination by EIPs and delayed the onset of contamination events, particularly in plumbing segments resistant to conventional cleaning. Foamed disinfectant interventions represent a promising strategy to mitigate the risk posed by sink drains in the healthcare environment. Future studies should investigate the long-term sustainability, cost-effectiveness, and clinical impact of foamed disinfectant interventions.
